# Evolution of Dental Resin Adhesives—A Comprehensive Review

**DOI:** 10.3390/jfb16030104

**Published:** 2025-03-14

**Authors:** Waad Khalid Alomran, Mohammed Zahedul Islam Nizami, Hockin H. K. Xu, Jirun Sun

**Affiliations:** 1 ADA Forsyth Institute, Cambridge, MA 02142, USA; 2 Harvard School of Dental Medicine, Harvard University, Boston, MA 02115, USA; 3 Department of Biomaterials and Regenerative Dental Medicine, University of Maryland School of Dentistry, Baltimore, MD 21201, USA

**Keywords:** dental adhesives, resin composites, TEG-DVBE

## Abstract

This comprehensive review of dental resin adhesives explores their historical development, key components, recent innovations, and potential future directions, highlighting a dynamic and continually advancing field. From Buonocore’s breakthrough acid-etching technique and Bowen’s pioneering dental resin invention, successive generations of clinicians and scientists have pushed forward the technological and materials development for secure bonding, while preserving dental tissues. The review discusses the substantial advances in improving adhesive reliability, enabling more conservative treatment approaches. It also delves into enhancing fundamental adhesive components and their synergistic combinations. Recent innovations, including biostable and functional resins, nanotechnology, and bioactive components, address persistent challenges such as durability, antimicrobial efficacy, and therapeutic functionality. Emerging technologies, such as digital dentistry, artificial intelligence, and bioinspired adhesives, portend an exciting and promising future for dental adhesives. This review underscores the critical role of ongoing research in developing biocompatible, multifunctional, and durable adhesives. It aims to support dental professionals and researchers by providing a comprehensive understanding of the dynamic progression of dental adhesives, inspiring continued innovation and excellence in restorative dentistry.

## 1. Introduction

Methacrylate-based resin composites were pioneered in the early 1960s by Dr. Rafael Bowen of the American Dental Association (ADA). The acronyms used throughout this proposal are listed in Appendix A. A decade later, these composites began to be widely used by clinicians to treat teeth affected by dental caries. Six decades on, methacrylate-based restoratives remain dominant in dental adhesives and restorative materials, owing to their natural tooth-like appearance, strong bonding capability with teeth, and versatility for small and large restorations. These unique properties have positioned them ahead of alternatives such as amalgam and glass ionomer cement [1,2,3].

Modern dental resin composite restoratives, along with their adhesives for dentin and enamel, typically consist of three essential components: (1) a resin network, (2) reinforcing filler particles, and (3) functional additives. Clinicians and researchers blend these components—like artists mixing colors—to address a wide range of complex dental cases. In addition, new materials and advanced technologies are consistently emerging, owing to more than fifty years of clinical experience and ongoing development [4,5,6,7].

Dental resin adhesives have emerged as foundational tools in restorative dentistry. Their development marked a pivotal shift from traditional mechanical retention methods to advanced adhesive techniques that enabled micromechanical and chemical bonding to dentin and enamel [8,9]. This transition has enabled more conservative treatment approaches, as dental adhesives minimized the removal of natural tooth structure and preserved more healthy tissue [1]. Additionally, by strengthening the bond between restorative materials and tooth surfaces, adhesives have enhanced the longevity and success of restorations, ultimately benefiting patients through increased comfort and reduced likelihood of restoration failure. This literature review provides an in-depth exploration of evolution, significant advancements, and the current state of dental adhesives. By highlighting their crucial role within dental science and projecting their future potential, the review underscores the transformative impact of adhesives on clinical practice and patient outcomes.

To gain a thorough insight into these developments, we conducted a systematic search that was conducted across multiple databases, including PubMed, Sco-pus, Web of Science, ScienceDirect, and Google Scholar, focusing on peer-reviewed articles published up to 2024 without a specific starting date, ensuring the inclusion of foundational studies to provide historical context. The search employed Boolean combinations of relevant keywords and MeSH terms, including “dental resin adhesives”, “adhesive dentistry”, “bioactive adhesives”, “self-etch adhesives”, “universal adhesives”, “bond strength of adhesives”, “composite restoration failure”, “remineralizing dental adhesives”, “antibacterial dental adhesives”, “nanotechnology in adhesive dentistry”, “polymerization shrinkage”, “hydrolytic degradation of adhesives”, “AI and machine learning in adhesive dentistry”, “digital dentistry adhesive bonding” and “clinical performance of adhesive restorations.” Articles were selected based on inclusion criteria, prioritizing clinical trials, systematic reviews, meta-analyses, and experimental studies evaluating adhesive performance, biomaterial innovations, antibacterial and remineralizing properties, digital dentistry applications, and AI-driven material advancements. Exclusion criteria were applied to eliminate non-peer-reviewed sources, conference abstracts, duplicated studies, non-English articles without translations, and publications lacking quantitative data or scientific validation.

Through our analysis, several key themes emerged over the development of adhesives from rudimentary to highly specialized formulae; continuous compositional improvements in search of better bond strength and biocompatibility; the addition of functional agents to tackle some clinical challenges; how to overcome deficiencies of existing materials; and the introduction of new concepts that could change the paradigm for dental adhesive applications soon [10,11,12,13].

This review first outlines the historical trajectory of dental adhesives, highlighting pivotal advancements in dental material science that have met the evolving demands of clinical practice. It then delves into the primary components of contemporary adhesives, such as resins and fillers, discussing their respective advantages and the practical challenges they address. Following this, we evaluate emerging alternative materials and technologies that show significant promise in addressing current limitations and advancing the science of dental materials. Finally, we offer a forward-looking perspective on anticipated trends in dentistry, focusing on research advancements and practical applications in clinical settings. Through this comprehensive review, we aim to assist dental professionals and researchers in understanding the dynamic progression of dental adhesives, thereby inspiring continued innovation and excellence in the field.

## 2. Historical Development of Dental Resin Adhesives

The trajectory of dental resin adhesives, integral to modern restorative dentistry, is rich with incremental advancements and innovations. Tracing back to its roots, the historical development of these materials reflects the progress in dental material sciences and the evolving needs of clinical practice.

### 2.1. Early Beginnings

The early beginnings of dental adhesives are to improve the retention of restorations within the oral cavity. Before the mid-20th century, dental restorative materials focused predominantly on mechanical retention. The principle was to design restorations physically locked into tooth structures, often necessitating extensive tooth preparation. This approach, while adequate to an extent, had several drawbacks, including removing healthy tooth tissue and potentially weakening the tooth structure.

Various materials, including zinc phosphate and silicate cement, were designed and tested in the early stages to enhance adhesion to tooth structures. However, these materials lacked bond strength and durability for long-term restoration success [14]. The search for improved adhesives led to the exploration of synthetic resins in the 1940s. Initially, these resins, such as polymethyl methacrylate, were used as filling materials due to their aesthetic properties and ease of manipulation. However, their adhesion to tooth structures was inadequate, leading to marginal leakage and secondary caries. It was not until Buonocore introduced the acid-etch technique in 1955 that a significant leap was made in adhesive dentistry. By etching the enamel with phosphoric acid, Buonocore created a roughened surface that enhanced the mechanical retention of acrylic resins. This technique represented the first step towards the adhesive revolution in dentistry, setting the foundation for future advancements in the field [15].

The early development of dental resin adhesives was thus characterized by a gradual shift from mechanical retention strategies towards a more adhesive approach. These initial steps set the stage for extensive research and development, leading to the sophisticated adhesive systems used in contemporary dentistry.

### 2.2. Buonocore’s Acid-Etch Technique

The inception of Buonocore’s acid-etch technique in 1955 marked a significant breakthrough in adhesive dentistry. This technique involved the application of phosphoric acid on enamel surfaces and enhanced adhesion between restorative materials and tooth structure by creating micro-roughness for mechanical interlocking [15]. This seminal development improved the durability and efficacy of dental restorations and laid the groundwork for subsequent advancements in dental adhesives. The acid-etch technique introduced a new paradigm by demonstrating that chemical bonding could be more effective. By etching the enamel with phosphoric acid, Buonocore achieved an increased surface area for bonding, significantly improving the retention of acrylic-based restoratives [16]. This technique effectively addressed the limitations of existing restorative procedures, such as marginal leakage and secondary caries.

Buonocore’s work inspired further research into the nature of adhesion to both enamel and dentin. The success of the acid-etch technique underscored the potential of developing adhesive systems that could bond to the different substrates in the oral environment. The introduction of Bis-GMA resin by Bowen in 1962 marked a significant breakthrough. This resin, compatible with etched enamel surfaces, formed stronger and more durable bonds.

In the subsequent years, enamel etching was extended to dentin. Early attempts to bond to dentin were challenged due to its complex structure. However, by the 1980s, breakthroughs in understanding the histology and physiology of dentin led to the development of dentin bonding agents that could penetrate the collagen matrix and form stable bonds [17].

### 2.3. Discovery of the Hybrid Layer

The discovery of the hybrid layer represents a pivotal development in the evolution of dental adhesives, fundamentally enhancing our understanding of dentin bonding. This breakthrough, primarily attributed to the work of Nakabayashi and his team in the early 1980s, has profoundly impacted the field of adhesive dentistry [17,18].

The hybrid layer, a microscopic zone at the interface between the adhesive resin and dentin, was first described by Nakabayashi et al. in 1982 [18]. They observed that, when dentin is treated with phosphoric acid and then infiltrated with resin, a unique intermingling of demineralized collagen fibers and resin occurs. This zone, termed the hybrid layer, is crucial in establishing a strong bond between the resin material and dentin substrate [17,19]. The hybrid layer’s effectiveness lies in its ability to provide micromechanical interlocking that significantly improves bond strength and durability—characterizing the hybrid layer led to a deeper understanding of the complexities involved in bonding to dentin, which is inherently more challenging than bonding to enamel due to its organic composition and moisture content. The hybrid layer formation was shown to be crucial for creating practical and durable bonds in restorative procedures involving dentin [19,20].

**Figure 1:**
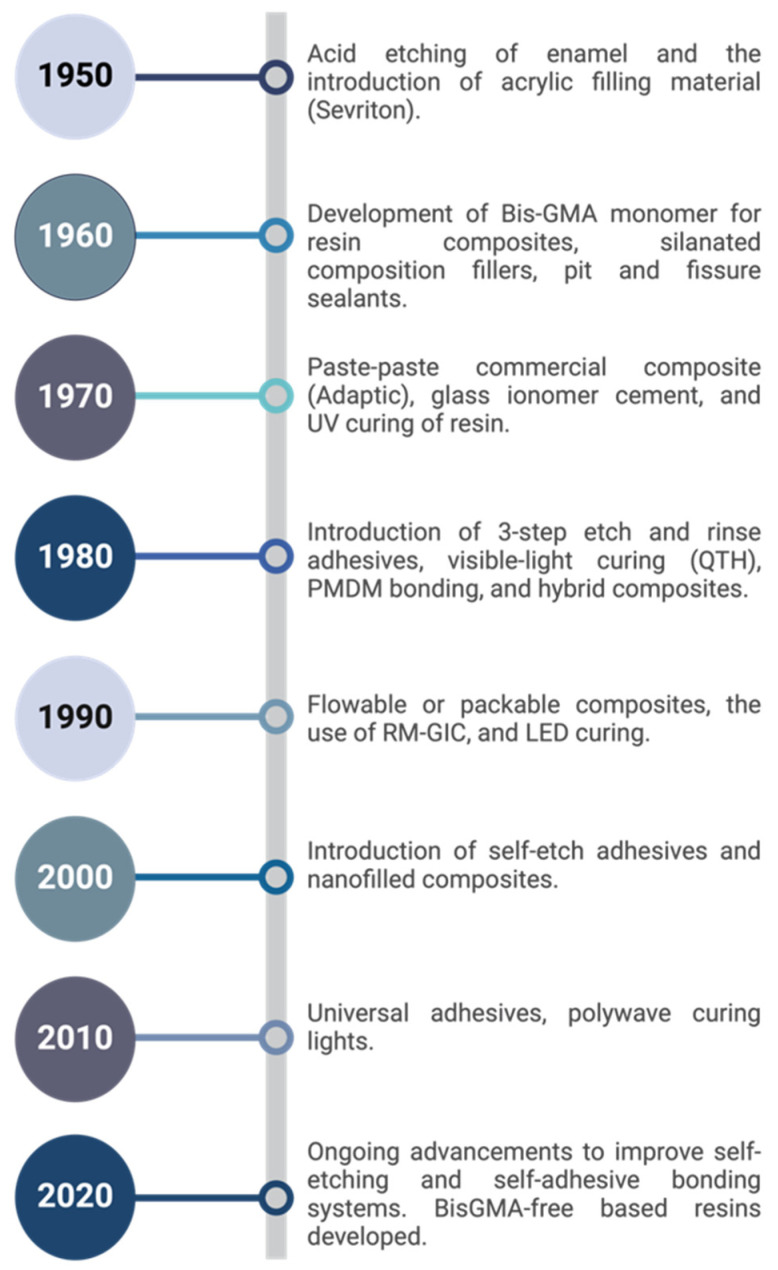
Timeline of milestones in dental resin adhesive development [3,15,23,24,25,26,27,28,29].

Subsequent research by Fusayama et al. in 1979 [21] and Gwinnett in 1991 [22] further elucidated the properties and formation mechanisms of the hybrid layer. These studies highlighted the importance of proper etching and resin infiltration techniques in achieving optimal hybrid layer formation, influencing the development of new adhesive systems and application protocols. The discovery of the hybrid layer also influenced the shift towards more conservative restorative techniques. It allowed for the preservation of more tooth structure by enhancing the bonding efficacy to dentin, thus supporting the principles of minimally invasive dentistry [2]. Figure 1 demonstrates the significant milestones in dental adhesive development.

### 2.4. From Total-Etch to Self-Etch Adhesive Systems

The progression from total-etch to self-etch adhesive systems in dentistry is a story of significant advancements in bond strength and the development of newer adhesive generations, each tailored to meet the evolving demands of clinical practice.

**Total-Etch Systems:** Originating from Buonocore’s acid-etch technique, total-etch systems (fourth and fifth generations) involve a phosphoric acid application to both enamel and dentin, followed by a bonding agent. These systems typically achieve bond strengths ranging from 20 to 30 MPa, providing adequate adhesion but requiring careful techniques to avoid issues such as postoperative sensitivity. Despite their effectiveness, total-etch systems are more sensitive to contaminants like saliva and blood, affecting their bond strength [30,31,32].
**Self-Etch Systems:** By contrast, self-etch adhesives (sixth, seventh, and eighth generations) integrate the etching and priming steps. These systems have bond strength values ranging from 18 to 35 MPa. They offer the advantage of reduced technique sensitivity and lower risk of postoperative sensitivity. Two-step self-etch adhesives have demonstrated higher shear bond strength than total-etch and multimode adhesives [33]. However, total-etch systems can exhibit higher bond strength in specific applications, such as when bonding to calcium silicate-based cement [34,35].
**Generational Progression and Comparison:** Each generation of dental adhesives has aimed to improve bond strength and application ease. The fourth and fifth generations (total-etch) focused on maximizing bond strength but were more technique-sensitive. In contrast, the sixth to eighth generations (self-etch) emphasized ease of use and consistency in performance, often with slightly lower but more predictable bond strength. Recent developments, like universal adhesives, seek to combine the strengths of both systems, offering versatility in application as they can be used as total-etch or self-etch and enhanced performance across diverse clinical scenarios [36,37].

In summary, the shift from total-etch to self-etch adhesive systems has marked a significant advancement in adhesive dentistry. While total-etch systems offer high bond strength under optimal conditions, self-etch systems provide consistent results with reduced sensitivity to technique and moisture.

### 2.5. Current Generations of Dental Adhesives

**First Generation of Bonding Agents:** A Swiss chemist, Oskar Hagger, who worked for DeTrey/Amalgamated Dental Company in the late 1940s, developed the first dental adhesive agent, Sevriton Cavity Seal [38]. This bonding agent had glycerolphosphoric acid dimethacrylate (NPG-GMA), and it was claimed to penetrate the dentin surface and prepare the surface for the chemically cured resin Sevriton [39]. Today, we call the resin-penetrated zone the hybrid zone/layer. These bonding agents preformed an ionic bond with the hydroxyapatite or a covalent bond to the collagen with a (hydrogen-bonding). However, this adhesive product had an overall poor clinical performance due to the high interfacial stress and thermal expansion caused by the methacrylate composites [40]. The bond strength was in the 1–3 MPa range [41]. The advent of first-generation dental adhesives had a profound impact on dental practices. It allowed for more conservative restorations since adhesives could effectively secure materials to a tooth structure with minimal preparation. This was a significant advancement over more invasive techniques requiring extensive tooth modification [42,43].
**Second Generation of Bonding Agents:** The second generation was introduced in the late 1970s and utilized an ionic bond between calcium and chlorophosphnate groups. These adhesives primarily used polymerizable phosphonate added to Bis-GMA resins to promote bonds to calcium ions [41,44]. However, this ionic bond was susceptible to degradation, even by the water within the dentin structure, resulting in microleakage when submerged in water. This generation did not remove the smear layer, resulting in a weak, unreliable bond strength [41]. This generation is no longer used due to its bonding failures with the loosely bonded smear layer. Also, the presence of water in the formula led to concerns about hydrolytic stability and degradation over time [44,45]. The second-generation adhesives marked the beginning of incorporating primers into adhesive systems, which later evolved into the more sophisticated multi-step systems of the third generation [22]. They also helped set the stage for the development of aesthetic dentistry, as these adhesives were more compatible with the translucent properties of newer composite materials.
**Third Generation of Bonding Agents:** This generation was introduced in the late 1970s and early 1980s. A revolution in this system was introducing the “total-etch” system to modify or partially remove the smear layer [41,46]. This allowed the penetration of the primer within the dentinal tubules after the acid was rinsed entirely away. Then, the primer will be added to the cavity, and an unfilled resin will be placed on dentin and enamel. The chemical composition of the primers and adhesives allowed for better interaction with the hydrophilic dentin and hydrophobic resin materials, improving the interface strength [19]. The weakness of this generation was the unfilled resins that did not infiltrate the smear layer [47]. While third-generation adhesives significantly improved bonding effectiveness, they were not without drawbacks. The total-etch technique increased the risk of post-operative sensitivity due to over-etching or incomplete sealing of dentin tubules. Additionally, the reliance on multiple application steps still posed a challenge regarding technique sensitivity [45]. Developing third-generation adhesives was a critical step towards the later introduction of simplified systems, such as the fourth and fifth generations, which combined the etching and priming steps or even included all steps in one application [48].
**Fourth Generation of Bonding Agents:** Introduced in the 1980s and 1990s, fourth-generation adhesives were developed to optimize the bonding process by separating the etching, priming, and bonding steps. This generation is often considered the gold standard for dental adhesive systems due to its high efficacy and predictable results [3,41]. The total-etch technique used with these adhesives involved phosphoric acid to etch both enamel and dentin, which provided a more uniform etch and a reliable bonding surface. This generation protocol was to remove the smear layer entirely, and it is still considered the golden standard. It has three primary components: an acid etchant, a primer, and a bonding agent. These systems are very effective when correctly used as they are technique -sensitive. The enamel and dentin had to be etched with phosphoric acid for 15–20 s and then rinsed, and the surface must be left moist to avoid collagen collapse. Using a hydrophilic primer enhanced the infiltration into the collagen network and formed the hybrid layer [41]. Due to hybridization, the bond strength improved significantly compared to previous generations. It ranged from low to mid-20 MPa [15]. On the downside, these systems can be time-consuming and confusing with many bottles and application steps.
**Fifth Generation of Bonding Agents:** The fifth generation of dental adhesives, which appeared in the late 1990s, introduced single-bottle systems combining the primer and adhesive in one solution. This generation aimed to simplify the bonding procedure while maintaining the high bond strengths of the fourth-generation systems. They are known to be the “one-bottle” or “one-step” systems [42]. The composition typically included a mixture of hydrophilic and hydrophobic monomers, solvents such as ethanol or acetone, and photoinitiators in a single solution. The consolidation into one bottle aimed to reduce variability in application and decrease the potential for technique-sensitive errors. The primer was combined with the bonding agent into one solution to be applied simultaneously after acid etching with 35–37% phosphoric acid, and this technique prevented collagen collapse and minimized postoperative sensitivity [41,45,49,50]. The single bottle etch-and-rinse adhesive type shows the same mechanical interlocking and comparable bond strengths to the 4th generation. However, they faced criticism for potential compromises in bond strength compared to their predecessors, particularly regarding long-term durability and susceptibility to hydrolytic degradation. They were more prone to water sorption and degradation over time than the 4th generation [51,52].
**Sixth Generation of Bonding Agents:** The sixth generation was introduced in the latter part of the 1990s and early 2000s. A self-etching primer is applied to the tooth surface, followed by a bonding agent. The concept of self-etching was first introduced in a publication by Watanabe and Nakabayashi in 1993 [53]. The most significant advantage of this system is that it is less dependent on the hydration status of the dentin than the total-etch systems. Innovations in sixth-generation adhesives have focused on enhancing the chemical composition of the primers to improve their efficacy and compatibility with both enamel and dentin. Specifically, the introduction of functional monomers, such as methacryloyloxydecyl dihydrogen phosphate (MDP), enhanced the adhesive’s interaction with hydroxyapatite in the tooth structure. These modifications aim to improve the hybrid layer’s mechanical properties and increase the bond’s durability [54]. These systems form a bond that is stronger to dentin than enamel, which might be because their pH is not acidic enough to etch enamel [32]. To overcome this problem, it is recommended to utilize selective etching of enamel [51].
**Seventh Generation of Bonding Agents:** This system was introduced in late 1999 and early 2005. The primary innovation of seventh-generation adhesives lies in their all-in-one application, significantly reducing procedure time and the potential for error associated with multiple-step systems. These adhesives use an acidic monomer that demineralizes and infiltrates the tooth substrate, creating a bond in one step [35,51]. These are considered acidic systems, and they are prone to hydrolysis and chemical breakdown [55]. In addition, they are more hydrophilic than self-etching primer, making them more susceptible to water sorption, limiting the depth of resin infiltration into the tooth, and creating more voids [56]. These systems have the lowest initial and long-term bond strength of any adhesive in the market [57]. Shear bond strength of the 7th generation ranges from 19.80 to 30.30 MPa [58]. The convenience and speed of application have made seventh-generation adhesives particularly popular for quick and less invasive procedures. Their ability to effectively bond in a moisture-rich environment makes them suitable for pediatric dentistry and for patients with limited cooperation [37]. Research continues to focus on enhancing the formulation of these adhesives by optimizing the ratio of acidic monomers and solvents, and by developing new monomers that can provide stronger and more durable bonds [59].
**Eighth Generation of Bonding Agents:** Eighth-generation dental adhesives were introduced in the 2010s, and they are also known as “universal adhesives”, “multi-mode”, or “multi-purpose” because they may be used as self-etch (SE) adhesives, etch-and-rinse (ER) adhesives, or as SE adhesives on dentin and ER adhesives on enamel (a technique commonly referred to as “selective enamel etching”) [37]. As medical devices, universal adhesives are designed to bond to various substrates, such as enamel, dentin, and restorative materials, regardless of the application mode. However, their clinical performance depends on the chosen mode: the SE mode involves simultaneous etching and priming without a separate rinsing step, while the ER mode requires a separate phosphoric acid etching step followed by rinsing and applying the adhesive. Voco America introduced Voco Futurabond DC as the 8th generation of bonding agents in 2010. It contained nanosized fillers, which increased the monomer penetration and the hybrid layer thickness, providing better enamel and dentin bond strength, stress sorption, and longer shelf-life [60]. Shear bond strength of the 8th generation ranges from 22.10 to 37.10 MPa [58].

The primary innovation in eighth-generation adhesives is the inclusion of functional monomers such as 10-MDP, which chemically bond to tooth structure, and silane, which can bond to ceramic surfaces, making these adhesives suitable for various restorative materials. Using these multifunctional monomers simplifies the procedure while maximizing the bond strength. 10-MDP has mild-etching properties. It forms a stable ionic bond with calcium salts within the tooth structure. This bond was first proven by Yoshida et al. in 2004 using XPS (or X-ray photo-electron spectroscopy) [2]. In addition, a silane-containing universal bonding agent enhances adhesion between restorative materials (like composite resins and ceramics) and tooth structures by incorporating silane, which chemically bonds with silica-based ceramics and resin adhesives. These versatile adhesives work with various materials and etching techniques, improving bond strength, moisture tolerance, and reducing technique sensitivity for both direct and indirect restorations.

A systemic review and meta-analysis published in 2015 by Rosa et al [55]. concluded that selective etching of enamel before the application of a mild universal bonding agent improved the bonding integrity and durability, while prior etching of the dentin with phosphoric acid did not improve the bond strength and increased the risk of post-operative sensitivity [2,61]. In addition, universal adhesive systems showed the least cell cytotoxicity when compared to other systems [62,63]. Continuous advancements are being made to optimize the formulations of these adhesives to enhance their performance and ease of use. Recent innovations focus on improving solvent systems and photo initiators to enhance polymerization effectiveness and reduce technique sensitivity [64].

Table 1 summarizes the primary compositions and techniques of these eight generations. It also highlights their bonding mechanism and range of bond strength. In addition, the challenges of each generation and representative commercial products are listed. Although both the 7th and the 8th generations are “All in one bottle”, there are key differences between them that are represented in Table 2.

## 3. Fundamental Composition of Adhesive Materials

A traditional dental resin adhesive system includes etchants, primers, resin monomers, initiators, solvents, reinforcing fillers, and sometimes other functional ingredients such as antimicrobial agents [72,73,74]. This section focuses on the materials that make primers and adhesives in current commercially available dental resin adhesives. Methacrylate-based resins are the most popular contemporary dental primers and adhesives for direct restorations [75,76]. Table 3 lists the most common commercially available resin formulations. Additionally, we discuss the major components of dental resin adhesives, including base monomers, diluting monomers, initiators, and fillers. Given their increasing popularity, we specialize on adhesion-promoting functional monomers, vital components of 8th-generation dental adhesives. However, clinical evidence has highlighted certain shortcomings of these contemporary dental adhesives. We will also discuss these drawbacks, emphasizing the need to develop a new generation of dental adhesives.

### 3.1. Common Composition of Current Dental Resin Adhesives

#### 3.1.1. Matrix Resins

Matrix resins are critical components in dental adhesives, providing the necessary structural framework that binds the fillers and transfers stresses within the adhesive. The matrix resins determine the adhesive system’s mechanical properties, durability, and clinical efficacy.

**Base Monomers:** Base monomers form the skeleton of the resin, determining its physical properties, such as flexural strength and compressive resistance. Two commonly used base monomers include Bisphenol A-Glycidyl Methacrylate (Bis-GMA) and Urethane Dimethacrylate (UDMA). These monomers provide excellent mechanical properties. However, these monomers must often be modified or diluted with other components due to their high viscosity to improve handling. For instance, Bis-GMA offers high strength and rigidity but requires dilution to enhance flow and manipulation properties [104]. Similarly, UDMA is used for its flexibility and toughness but requires diluting agents to achieve optimal handling properties [1,105].
**Diluting Monomers:** Diluting monomers adjust dental resins’ viscosity and handling properties. These monomers are less viscous than base monomers, allowing for easier resin application in clinical settings. Diluting monomers such as TEGDMA or HEMA are incorporated into the formulation to reduce the viscosity of the resin, making it easier to handle and apply. In 1-step self-etching adhesives to prevent phase separation between water and the adhesive monomers [106]. In addition, they are vital in optimizing the degree of polymerization and conversion, affecting the material’s shrinkage and overall mechanical performance. However, their inclusion must be carefully balanced, as excessive use of diluting monomers can weaken the mechanical properties of the resin, potentially leading to reduced restoration longevity [4,107].

#### 3.1.2. Initiators

Dental resin adhesives rely on initiators to catalyze the polymerization of resins into solid matrices that bond restorative materials to tooth structures. These initiator systems are categorized into three primary types: chemical-cure, light-cure, and dual-cure systems. The choice of initiator affects the adhesives’ properties, including degree of conversion, mechanical strength, and aesthetic stability. The amount of initiator added to adhesive systems depends on the type of initiator and the adhesive system but is usually in the range of 0.1–1 wt% [62].

**Chemical Initiator Systems:** Chemical initiator systems, also known as self-cure or auto-cure systems, rely on a catalyst (usually benzoyl peroxide) and an activator (commonly an amine) to initiate polymerization without light. Valid areas where light cannot penetrate, such as deep cavities or post-core build-ups [13]. However, it has a shorter working time and potential for color instability [1,86,108].
**Photo-Initiator Systems:** Photo-initiator systems use light to activate polymerization. The most common initiator is camphorquinone (CQ), which is activated by blue light. CQ is generally used with an amine co-initiator to accelerate the production of free radicals for polymerization. CQ/amine combination initiates polymerization upon exposure to visible light at approximately 465 nm [90]. However, CQ’s yellow color may affect aesthetics [90]. Combining CQ with Ivocerin or Lucirin TPO broadens the absorption spectrum and improves the depth of cure. It improves esthetics and enhances the depth of the cure [4]. Phenylpropanedione absorbs light at a similar wavelength to CQ but with slightly different optical and polymerization properties. It may be used as a substitute or in combination with CQ to reduce yellowing effects [86].
**Dual-Curing Systems:** Dual-cure systems are designed to polymerize through both chemical and light-initiated mechanisms. These systems are beneficial when light penetration is limited, such as in deep cavities or beneath opaque restorations [109]. The most popular combination in dual cure systems is CQ with a tertiary amine for the light-curing component and benzoyl peroxide with an aromatic sulfonic acid salt for the chemical-curing component [13,86].

#### 3.1.3. Fillers

Fillers are particularly noteworthy as they improve the adhesive’s mechanical strength, viscosity, and handling characteristics. Fillers influence the adhesive’s properties, including polymerization shrinkage, wear resistance, radiopacity, and handling characteristics. Silica fillers are among the most widely used traditional fillers in dental adhesives. They may be silanized and provide improved bonding in the resin matrix. However, these silanization agents are generally methacrylate-based compounds subject to hydrolytic degradation in the oral environment [110]. Traditional dental adhesives often incorporate aluminosilicate glass fillers, contributing to the material’s structural integrity and caries prevention through fluoride release [111]. These fillers have been used for many years due to their strength and anti-cariogenic properties. In older formulations, zinc oxide-polyacrylic acid-based fillers in dental cement show some adhesion to tooth tissues. However, they are often replaced by more advanced composite materials today [112].

### 3.2. Adhesion Promoting Functional Monomers in Dental Resin Adhesives

Functional monomers play a critical role in the bonding efficacy of dental adhesives, especially at the interface between the adhesive and tooth structure. Functional monomers establish chemical interactions with tooth tissue components. They are pivotal in achieving strong and durable bonds, which are crucial for the long-term success of dental restorations. Studies have shown that functional monomers, such as phosphate monomers 10-MDP and MF8P, the phosphate functional group, can form strong ionic bonds with hydroxyapatite. They improve bond durability in dental adhesives, ensuring the longevity of restorations [62,113] owing to the low solubility of the resulting calcium salts. Functional monomers also etch the enamel and dentin surfaces in self-etch adhesive systems. The impact of functional monomers in all-in-one adhesive systems on the formation of enamel/dentin acid-base resistant zones was investigated by Nikaido et al. Variations in thickness and morphology were observed between enamel and dentin interfaces, influenced by the specific functional monomers used in the adhesive systems [114]. For example, 10-MDP is one of the mild acidic monomers that enable a universal adhesive to be used with any etching technique in a pH ranging 2–3 [115]. This compound was invented by Kuraray (Osaka, Japan). Its dihydrogenphosphate group can dissociate in water and establish an intensive and stable chemical interaction with hydroxyapatite [116].

**Figure 2:**
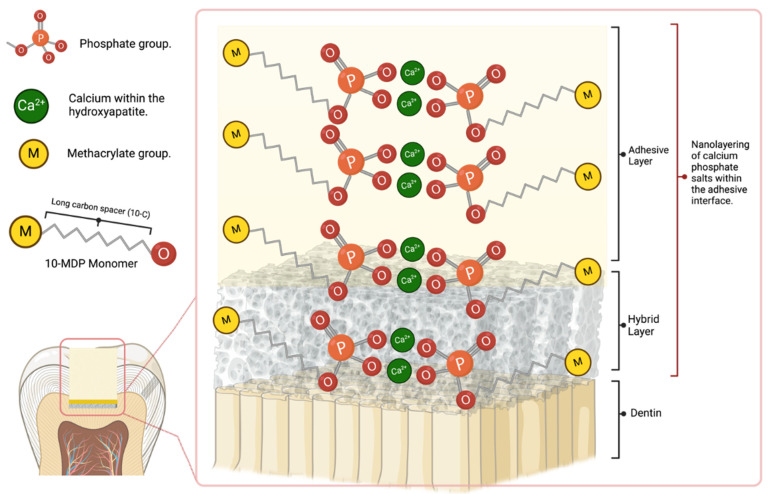
Schematic explaining the formation of MDP-Ca salt and interfacial nanolayering.

Stable MDP-calcium salts are formed during this reaction and deposited in self-assembled nano-layers, illustrated in Figure 2 [117]. Nano-layering of 10-MDP_Ca salts was documented to still exist after 1 year of water storage, and it was found to be aging-resistant due to the low dissolution rate [97,118]. This ionic bond makes the adhesive interface more resistant to biodegradation [37,67,116]. The bonding strength, e.g., micro-tensile bond strength (μTBS), was maintained after 1 year of aging [119]. The good in vitro and clinical outcomes of the Clearfil SE Bond, an adhesive containing 10-MDP, may be partly attributed to its intense chemical adhesion with tooth tissue [4]. It was proven that 10-MDP or 10-MDP-Ca salt inhibits the matrix metalloproteinases (MMPs) that accelerates the degradation of the hybrid layer, which in turn reduces nanoleakage of the bonding interface and improves its durability [120]. Van Landuyt et al. considered the most promising monomer for chemical adhesion to the hydroxyapatite to be 10-MDP. Feitosa et al. (2014) studied the influence of spacer carbon chains in acidic functional monomers on self-etch dental adhesives’ physical and chemical properties. They found that highly hydrophilic monomers with longer spacer carbon chains increased wettability and water sorption on dentine surfaces, affecting the ultimate tensile strength of one-step self-etch adhesives [121].

However, combined use of 2-hydroxyethylmethacrylate (HEMA) and acidic functional monomer may compromise the bonding. Yoshida et al. (2012) [98] investigated the effect of 2-hydroxyethylmethacrylate (HEMA) on the nano-layering of the functional monomer 10-MDP at the interface with hydroxyapatite-based substrates. They discovered that adding HEMA inhibits the nano-layering of MDP at the interface and subsequently compromises the long-term durability of the bond to tooth tissue. In addition, using these acidic monomers may reduce the shelf-life of methacrylate-based resin adhesive due to ester hydrolysis. They also affect the polymerization efficiency, mainly when amine is used as the initiator [98].

### 3.3. Challenges of Current Dental Adhesives

There have been ongoing advances in adhesive dentistry for decades, particularly in the various elements of composition and classification. However, optimal performance has yet to be developed for direct adherence, bond strength, stability, and durability. Most commonly, adhesive interface failure results in clinical complications like sensitivity, stains, and secondary caries that ultimately compromise the restoration [122]. Current research has identified some of the following factors that affect the adhesive-dentine interface.

#### 3.3.1. Biocompatibility and Toxicity of Dental Resins

Recently, the biocompatibility of resin monomers has come under significant scrutiny. Research indicates that residual monomers can leach into saliva after curing, and resin degradation can lead to additional chemical release into the human body. Many monomers and degraded products, particularly methacrylate-derivatives, have been found to exhibit cytotoxic effects. In addition to their cytotoxicity, concerns have been raised about the potential endocrine-disrupting effects of these monomers [123,124,125,126,127].

Despite the widespread use of Bisphenol A-glycidyl methacrylate (Bis-GMA) resin in dental composites, there are concerns about its potential toxicity, primarily due to its potential to leach out of the composite material over time. The main concern with Bis-GMA is BPA, a substance that has been linked to a variety of health problems, including hormonal disruptions and potential carcinogenic effects [82,83,128,129,130,131,132]. When Bis-GMA is used in dental composites, it can potentially release BPA as the initial raw materials might contain unreacted BPA or degrade over time [133].

Once released, BPA can be swallowed or absorbed into the oral tissues, leading to systemic exposure. A recent study showed absorption of BPA by the sublingual area in dogs, allowing its direct entry into the bloodstream, bypassing the digestive system and liver, and multiplying its bioavailability by a factor of 80 [134]. While the exact health effects of this exposure are still being studied, there is concern that it could contribute to hormonal disruptions or other health problems due to the estrogenic activity of BPA [129,130,132,135,136]. More research has reported the health effects of Bis-GMA. Kuan and his colleagues conducted an experiment that showed Bis-GMA demonstrated cytotoxicity to macrophages in a dose- and time-dependent manner. It also induced the production of Prostaglandin E2, a key regulator of immunopathology in inflammatory reactions [137]. Several additional studies were performed by Sadeghinejad et al., aimed at investigating the impact of Bis-GMA biodegradation products on cariogenic bacteria. The researchers discovered that bishydroxypropoxyphenyl-propane (BisHPPP), a Bis-GMA biodegradation product, had a significant effect on the gene expression and protein synthesis of cariogenic bacteria, which could potentially result in increased secondary caries around resin composite restorations [138]. Another team study found that triethylene glycol, a hydrophilic biodegradation product of Bis-GMA, stimulated the growth of Streptococcus mutans, a dominant cariogenic bacterium, and up-regulated genes linked to bacterial virulence [139]. Bis-GMA is also considered cytotoxic, which can alter the cell cycle and induce oxidative stress, leading to apoptosis and necrosis in a concentration-dependent manner. High concentrations of Bis-GMA resulted in cell death by necrosis, while low concentrations resulted in cell death by necrosis or late apoptosis [140,141]. The degradation of Bis-GMA and release of BPA can occur due to several factors: (1) During the curing process, not all of the Bis-GMA may fully polymerize, leaving residual monomers that can potentially leach out over time. (2) Over time, the composite material can degrade due to wear and tear, as well as exposure to the oral environment (e.g., changes in pH, temperature, and exposure to bacterial and oral enzymes). (3) Exposure to certain solvents, such as alcohol or other components of oral hygiene products [81,83].

HEMA (2-hydroxyethylmethacrylate) is a hydrophilic methacrylate monomer that is frequently added to dental adhesives due to its wetting enhancement effect and promotes the diffusion of co-monomers by expanding the demineralized collagen [142,143]. Apart from the main disadvantage HEMA poses of facilitating water uptake and the subsequent gradual hydrolytic degradation of the polymers, swelling, and resin staining [55]. Spagnuolo et al. also reported on the cytotoxicity of HEMA as unreacted HEMA increased levels of reactive oxygen species (ROS). They decreased the levels of antioxidants like glutathione, causing oxidative dysfunction and apoptosis of fibroblasts in the pulpal tissues [144,145]. Lee et al. also reported on the cytotoxicity of HEMA as it induced cell toxicity in RAW264.7 macrophages with a concentration-dependent matter. A higher HEMA concentration was associated with a higher level of apoptosis and genotoxicity [146].

#### 3.3.2. Effects of Oral Conditions on Adhesive Durability

Oral bacteria metabolize dietary sugars and produce acids, primarily lactic acid, which challenges the stability of dental adhesives by lowering the pH at the adhesive-tooth interface. Lower pH accelerates the hydrolysis of the resin matrix and demineralization of tooth structure. This acidic environment poses a risk to both secondary caries, adhesive degradation, and eventually restoration failure [147].

Masticatory forces are unavoidable oral situations that affect the adhesive and tooth bonding interface. They usually impact adhesive bond strength and generate marginal leakage on the restoration surface. Furthermore, incomplete infiltration of adhesive triggers leakage formation. Simultaneously, formed leakages enhance the exposure of dentin collagen and accelerate oral fluid absorption, causing the degradation of adhesive and collagen matrix [148,149,150,151,152].

Generally, dentine’s intrinsic matrix protease activity is responsible for collagen degradation over time. At the same time, treated dentine with some enzyme inhibitors showed a notable decrease in collagenolytic and intrinsic dentine gelatinolytic activity [153,154,155]. Reports have shown that matrix metalloproteinases (MMPs) and cysteine cathepsins (CTs) are the best-known endogenous enzymes among all enzymes in dentine and are responsible for collagen degradation [156,157,158].

#### 3.3.3. Properties and Performance of Dental Resin Adhesive That Need to Be Improved

Hydrolysis of the ester functional groups in the resin’s constituent monomers was reported to contribute to resin matrix degradation. They are readily hydrolyzed and degraded by oral enzymes and cariogenic bacteria, incomplete infiltration of the resin monomers into the dentinal tubules, and the high-water absorption of the resin network [159,160,161,162]. Moreover, ester bonds in methacrylate adhesives are also prone to chemical hydrolysis [154,163]. Reports showed that water incorporated in a hybrid resin layer acts as a medium to initiate hydrolysis. It also plays its role as a plasticizer within the adhesive polymer chains, accelerating the matrix degradation [164,165]. Additionally, cholesterol esterase and pseudocholinesterase in saliva facilitate methacrylate’s degradation. On the other hand, hydrophilic and hydrophobic-containing dental adhesives experience a phase separation phenomenon that leads to degradation [166].

Incomplete infiltration usually leaves a microleakage caused by either inadequate adhesive dispersion or the lack of function of acid etch [167,168,169]. This microleakage exposed dentin collagen and facilitated the activity of MMPs and CTs. Therefore, the bonding strength of the adhesive is affected more readily than completely sealed dentine. [154,170,171] A firm and complete hybrid layer is complex in such a condition. At some point, monomers take up space at the interface, which cannot protect collagen-bonded fluid flow. On the other hand, large monomers (Bis-GMA) may become trapped in inter-fibrillary spaces and cannot penetrate the hybrid layer. However, small monomers (HEMA) may penetrate but provide frail linear chains, eventually resulting in the collagen’s cyclic fatigue failure [170].

Various factors, including polymerization shrinkage, inadequate bonding, incomplete tooth preparation or defragment of restoration area, inconsistent adhesives application, and other associated compression stresses such as natural wear and tear that occur over time, are responsible for microleakage. Oral fluids and microbes can invade this microleakage and penetrate the adhesive interface. The acid produced by cariogenic bacteria and bacterial collagenases enhances adhesive degradation, leakages, and lease [172,173,174,175]. At the same time, it activates MMPs and CTs activity and synergistically increases the degradation of adhesives and collagens, thereby reducing the stability and durability of the resin-dentin bond [176,177,178].

## 4. Advances in Solving Challenges

The dental research community has widely acknowledged these challenges. Notably, the Dental Materials Innovation Workshop held in London in December 2012, and its subsequent publication in Advances in Dental Research (2013), emphasized the critical need for hydrolytically stable resin networks in dental restorations [179]. In response, the National Institutes of Health (NIH) in the United States funded six projects to develop next-generation dental resin restoratives. This recognition and support have inspired scientists to innovate and create new materials designed to address these challenges, ultimately improving the performance and longevity of dental resin adhesives. In this section, we highlight several new materials and technologies that have been developed to meet these goals.

### 4.1. Hydrolytically Stable Resin Networks

Table 4 highlights recent advancements in methacrylate-based resins for dental adhesives, summarizing their enhanced properties and limitations as reported in the literature.

New monomers and polymerization mechanisms have been introduced as alternatives to hydrolyzable methacrylate monomers [206,207,208,209,210]. Among these advancements, a step-growth thiol-ene reaction has been proposed as a substitute for the conventional radical polymerization method [211,212,213,214]. This approach notably delays the gelation process, allowing higher degrees of conversion (DC), and reduces polymerization stress, a common cause of micro-leakage and tooth fractures. The high DCs achievable in these systems minimize the presence of unreacted monomers, significantly reducing the leaching of potentially toxic compounds [7,21,22,23]. Dental composites based on silorane, and those incorporating thiourethane oligomers, have demonstrated superior mechanical properties to traditional methacrylate-based composites [215,216,217]. Adding thiourethane oligomers further enhances the performance of resin composites [218,219].

**Figure 3:**
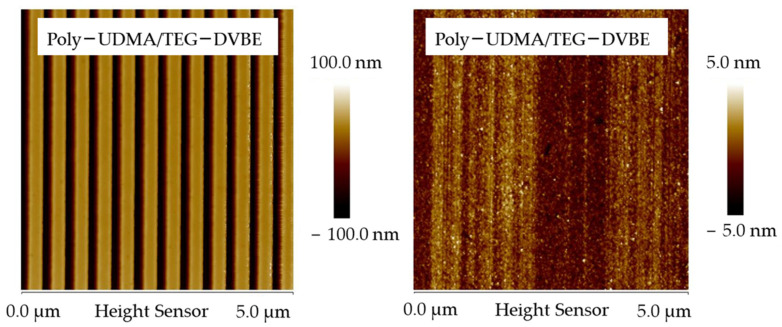
Biostability Comparison of Resin Networks. Atomic force microscope (AFM) scans illustrate the biostability contrast between a hybrid resin network, poly-UDMA/TEG-DVBE (**left**), and a methacrylate-based resin network, poly-UDMA/TEGDMA (**right**). Nano-scale line-and-space patterns (line-height: 110 nm; line width: 135 nm) were imprinted onto the two resin networks using nanoimprint lithography. The images show the patterns after exposure to the enzyme pseudocholine esterase for 3 days, highlighting the differential resistance to enzymatic degradation.

Gonzalez-Bonet et. al. successfully synthesized and characterized multiple ether-based monomers that may replace conventional resins. Specifically, triethylene glycol divinylbenzyl ether (TEG-DVBE) and its homopolymer showed no degradation under the challenges of PBS and esterase [195]. Yang et al. further discovered that the equimolar UDMA/TEG-DVBE mixtures were photo-polymerized in a controlled fashion. As a result, the styrene functional groups (ether-based TEG-DVBE) and methacrylate functional groups (ester-based UDMA) were packed alternatively. Such alternative packing protects ester functional groups from hydrolysis by hydrophobic styrene. The resulting copolymers are analogs of vinyl ester resin, a hybrid resin network made from copolymerization of styrene and methacrylate derivatives. The VERs are hydrolysis/corrosion-resistant materials that are superior to hydrolyzable polyesters. Moreover, they demonstrated the extraordinary biostability of poly-UDMA/TEG-DVBE in comparison to traditional ester-based co-polymers, poly-UDMA/TEGDMA using nanoimprint lithography Nano-scale line-and-space patterns (line height 110 nm; line width 135 nm) imprinted on poly-U/T were eradicated entirely within 72 h under esterase enzyme challenges (PCE 15 units/mL in PBS) while the same nanoscale patterns imprinted on poly-U/V are unaltered (Figure 3). The disappearance of nanopatterns on poly-U/T in only 3 days signifies the need to replace these hydrolyzable traditional resin networks. Furthermore, the distinguished poly-UDMA/TEG-DVBE biostability undeniably demonstrated its potential as a durable material in oral environments.

Ester-based and ether-based resins work differently in terms of stability and performance in dental applications. Ester-based resins (i.e., Bis-GMA) are commonly used for desirable properties, such as high viscosity and low volatility, that form a strong and durable polymer. However, ester groups are prone to hydrolysis in the moist oral environment. Hydrolysis leads to the degradation of the resin over time, which decreases its mechanical properties and durability and ultimately leads to the failure of the restoration [220,221]. Conversely, ether groups are resistant to hydrolysis. Therefore, they are more stable and durable in the oral conditions [221]. The report showed that hybrid resins containing both ether and ester groups have desirable properties when combined with both resins. The ester groups contribute to strength and durability, while the ether groups inhibit hydrolysis, enhancing the overall stability of the resin [195]. Introduction of TEG-DVBE (Triethylene Glycol Divinylbenzyl Ether), a monomer that forms a hydrophobic cross-linked polymer network, resists hydrolysis and water absorption. Thus, maintain the integrity of dental restorations in the moist oral environment [195].

### 4.2. Resin Matrix Reinforcement

Fillers, nanoparticles, and nanocomposites are widely considered to be used as reinforcing agents for adhesives [222]. The incorporation of carbon nanotubes [223], silicon dioxide nanoparticles [224], titanium oxide nanoparticles [225], and zirconia nanoparticles [226] were reported to improve the mechanical properties of adhesives. In addition, they facilitate elasticity on the adhesive-tooth interface that plays a role in counteracting stress caused by polymerization shrinkage.

### 4.3. Improving Adhesive Compatibility with Collagens

Reports demonstrated that adding MMPs and CTs inhibitors to adhesives improves the durability of adhesives [227]. Inhibitors reduce the degradation of the collagen fibrils [154]. Recently, various crosslinking agents were incorporated into adhesives to prevent the degradation of collagen fibers, where they were added as primers into the adhesive composition [228,229]. Chlorhexidine (CHX) [230,231,232,233], glutaraldehyde [234,235,236,237], and zinc-doped adhesives [238,239] were predominantly added to the dental adhesive to inhibit collagen degradation. At the same time, some natural anti-MMPs, CTs, and collagen crosslinking agents were also reported to be incorporated in the dental adhesive to prevent collagen degradation, such as grape seed extract (GSE) [240,241,242], hesperidin (HPN) [243,244,245], and quercetin [246,247,248]. Pretreatment of the restoration site was also reported to inhibit MMPs activity. Pretreatment is the last step before adhesive application on the restoration site. Based on required properties improvement various application agents were reported. Carbodiimides were found to be applied to inhibit MMPs [249,250]. Ethylenediaminetetraacetic acid (EDTA) was used to inhibit MMPs activity and remove the smear layers that reduce the chances of incomplete infiltration of adhesives. In addition, EDTA application enhances mechanical retention [251]. Galardin application was also reported to be used for the inhibition of MMPs activity [252]. Besides the incorporation of natural agents in adhesives, some of them were also reported to be used in the pretreatment of restorations for MMPs inhibition, including Epigallocatechin-3-gallate (EGCG) [253], grape seed extract (GSE), and quercetin [247].

### 4.4. Functionalization of Adhesives for Remineralization

The prevalence of secondary caries formation, incomplete penetration of adhesion, marginal leakage, and collagen degeneration always increases the risk of demineralization at the adhesive tooth interface. Ongoing research focuses on increasing the remineralization of the interface to serve sustainable restorations. Reports have demonstrated that the remineralization process replaces water from the interface of the hybrid layer with apatite deposition. Thus, it reduces hydrolytic activity and maintains strong bonds [254,255,256]. The functionalization of adhesive with remineralization agents benefits the stability of adhesive-tooth bonding, enhances mechanical properties, and prevents the exposure of MMPs and CTs [257,258]. Several agents were reported to be incorporated into adhesives to add remineralizing properties, including amorphous calcium phosphate nanoparticles (NACP) [259,260], bioactive glass (BAG) [261,262,263], Cu-doped BAG [258], dentin phosphoproteins analogs [264,265], and hydroxyapatite [266,267].

### 4.5. Providing Adhesives with Antibacterial Activity

The antibacterial effects of dental adhesives are advantageous in inhabiting residual bacteria in restoration surfaces. At the same time, it prevents secondary bacterial innovation in adhesive-tooth interface through marginal microleakage [268,269,270]. Adding an antibacterial ingredient to dental adhesive may have additional benefits against oral bacteria and biofilms that attack the adhesive-tooth interface, leading to resin degradation and secondary caries’ formation [271]. Chitosan [272], fluoride [273], quaternary ammonium salts [274,275], silver nanoparticles [276], and surface pre-reacted glass ionomers [277] have been tested in dental adhesives to add antimicrobial activity.

Recent dental adhesive developments incorporate various functional fillers and additives to enhance their properties. Carbon nanoparticles, chitosan, iron oxide, and titania nanoparticles improve bond strength, while copper and zinc oxide nanoparticles also reduce bacteria, biofilm formation, and enzymatic degradation, stabilizing the adhesive layer. Hydroxyapatite and wollastonite promote mineralization and bonding durability, while wollastonite also enhances mechanical properties over time [12,147,222,275,278,279,280,281,282,283,284,285,286,287,288,289,290,291,292,293,294].

Antibacterial agents such as silver nanoparticles, chlorhexidine, doxycycline, and others (e.g., benzyl dimethyl dodecyl ammonium chloride, triclosan) target biofilm and secondary caries prevention. Novel materials like boron nitride nanotubes (BNNTs) and graphene nanoplatelets show promise for mechanical stability and anti-biofilm effects. Pre-reacted glass ionomer (PRG) fillers enhance fluoride release and bonding longevity. Compounds like resveratrol and 4-formylphenyl acrylate also focus on improving bond strength, demonstrating the potential for future innovation in adhesive materials. Table 5 lists experimental fillers and additives used in the resin network to provide additional functions in dental resin adhesives.

The evolution of dental resin adhesives has significantly improved restorative outcomes, shifting from traditional mechanically retained materials to advanced chemical bonding systems. Other adhesives, such as zinc phosphate and glass ionomer cement (GICs), primarily relied on mechanical interlocking for retention, often necessitating extensive tooth preparation to create macro-retentive features. Although GICs offered fluoride release, their bond strength ranged between 5 and 15 MPa, which resulted in higher microleakage and increased secondary caries risk over time [325]. In contrast, modern adhesives, particularly self-etch and universal systems, incorporate functional monomers such as 10-MDP, which establish durable chemical bonds with hydroxyapatite, significantly improving bond strength to over 30 MPa [326]. Moreover, antibacterial monomers like MDPB and nanoparticle-reinforced adhesives have strengthened modern adhesive systems by inhibiting bacterial growth and promoting dentin remineralization, reducing the incidence of secondary caries and adhesive degradation [315]. Additionally, while traditional adhesives require precise moisture control and etching procedures, making them highly technique-sensitive, universal adhesives offer simplified, more predictable application protocols, allowing for self-etch, selective-etch, or total-etch approaches with improved clinical outcomes [79]. The transition from traditional to modern adhesives has resulted in higher bond strength, reduced microleakage, and enhanced ease of application, making universal and bioactive adhesives the preferred choice in contemporary restorative dentistry. Table 6 illustrates examples of market-available functionalized adhesives.

## 5. The Next Generation of Dental Adhesives

The field of dental adhesives is undergoing significant transformation, driven by emerging technologies, advanced biomaterials, and the integration of digital and AI-powered solutions. Innovative pretreatment techniques enhance adhesive performance, with universal adhesives, laser treatments, and novel bonding protocols offering improved bond strength, durability, and stability. Concurrently, advancements in biomaterials are addressing key challenges in resin restoratives, introducing multifunctional, biocompatible materials that mimic natural tooth structures while preventing degradation and secondary caries. As digital dentistry revolutionizes restorative workflows, dental adhesives adapt to ensure precise bonding for CAD/CAM and 3D-printed restorations. Meanwhile, AI and machine learning accelerate adhesive development by optimizing formulations, personalizing treatments, and predicting clinical outcomes, heralding a new era of intelligent and efficient dental care solutions.

### 5.1. Emerging Technologies in Pretreatment

Emerging technologies, particularly the development of universal adhesives, are a focus area. These adhesives are designed to function effectively across total-etch, self-etch, or selective-etch modes, offering versatility and enhanced bond strength. Simultaneously, researchers suggested modifications to existing protocols. Laser pretreatment was reported to be beneficial for adhesive application. Laser pretreatment to acid-etched dentin softens and occludes orifices of dentinal tubules and reduces dentin permeability [343]. At the same time, laser treatment of the bonding agent before curing improves the adhesive and tooth surface interface bonding. Moreover, increased temperature due to laser application was also reported to be beneficial for adhesive infiltration and evaporation of unwanted fluids and solvents [343,344]. Non-thermal atmospheric plasma treatment was also reported to increase hydrophobicity, thus enhancing the adhesive penetration and interfacial bond strength [345]. Some studies suggested modifying the bonding protocols. An approach known as Dimethyl Sulfoxide (DMSO) wet bonding was reported to improve adhesive-tooth interface stability. In this approach, DMSO facilitates the removal of water from dentin, thereby increasing the bond strength of etch-and-rinse and self-etch adhesives, even after aging. Additionally, DMSO can stabilize the demineralized dentin matrix, inhibit matrix metalloproteinases (MMPs), and improve adhesive infiltration. This protocol could be promising in achieving the desired adhesive-tooth bonding interface to incomplete infiltration and microleakage and preventing secondary caries, thereby encompassing the durability of adhesives [346,347,348]. In wet bonding, water can be a reason for phase separation. In addition, water-suspended dentin collagen acts as a barrier. As a result, incomplete infiltration may occur. Ethanol-wet bonding protocol was reported using ethanol instead of water in etch-and-rinse procedures for adhesive application. [349,350] This process prevents hydrolysis and inhibits MMP activities [351].

### 5.2. Advancements in Biomaterials

As the amalgam phases out, resin restorative becomes a promising replacement. The advanced biomaterials will be easy to apply, error-forgiving, biocompatible, and high-performance. These materials focus on biocompatibility while being functional and durable. They maintain the integrity of the collagen structure and enhance restorations’ aesthetic and functional outcomes [352,353].

Next-generation dental adhesives will be multifunctional, preventing the key challenges that induce secondary caries. These challenges include hydrolysis of the resin network, cariogenic bacteria attack, and stresses related to polymerization shrinkage and mechanical forces. In addition to the new hydrolytically stable resins discussed in the previous section, antimicrobial potential is desirable. Moreover, self-healing or self-repairing functions that may recognize microcracks and other defects and repair them autonomously without human intervention will be helpful. Innovative materials have been proposed to achieve self-healing functions through double network chemistry, self-healing design and bioactive fillers [354]. These new developments will also correct the suboptimal placement of resin restoration, thus significantly enhancing resin restorative’s applicability in underdeveloped areas with limited resources and inadequately skilled health professionals.

Biomimetic materials are the new era for designing and developing dental adhesives [355,356]. Biomimetic materials in dentistry are designed to mimic the natural structure and function of tooth tissues, such as enamel and dentin. These materials aim to replicate the mechanical, chemical, and biological properties of natural teeth to restore function and aesthetics while promoting integration with the surrounding tissues [357,358]. For example, mussels’ moisture-resistant adhesion properties and firm surface coating ability have impressed dental research. A recent study investigated mussel-functionalized catechol-thiol-based dental adhesives, a catechol-functionalized copolymer, poly (dopamine-methacrylate-co2-methoxyetheyl acrylate) (pDMA-MEA) was analyzed as a primer for an etch-and-rinse adhesive and reported to improve the bond strength in a saliva-contaminated condition [359]. Also, Biomimetic composites that mimic the layered structure of enamel and dentin [357,360,361,362,363].

However, controversies and challenges remain. Some studies question the long-term effectiveness of ion release and remineralization potential, as the bioactive properties may diminish over time [363,364]. Balancing bioactivity with mechanical strength and durability is another significant challenge, as these materials often exhibit inferior mechanical properties compared to traditional resins, limiting their use in high-stress areas [1,3]. Additionally, there is a need for more long-term clinical studies to validate the benefits and safety of bioactive adhesive resins, as current evidence is often limited to in vitro or short-term in vivo studies [365]. Addressing these challenges is crucial for widely adopting these materials in clinical practice.

### 5.3. Dental Adhesives in Digital Dentistry

Dental resin adhesives are critical in enhancing digital dentistry by ensuring strong and durable bonding for CAD/CAM and 3D-printed restorations [79,366]. The key characteristic of dental adhesives for digital dentistry is their versatility and reliability in bonding to a wide range of materials used in digitally fabricated restorations, such as ceramics, composites, and 3D-printed resins. These adhesives must provide strong and durable bonds, ensuring long-lasting adhesion and precise marginal sealing to prevent microleakage. They support fast curing times to streamline workflows for same-day procedures and offer aesthetic stability by maintaining color and translucency. Universal formulations simplify application, while bioactive or antibacterial properties enhance tooth health. Together, these features ensure efficient, precise, and predictable outcomes in digital dentistry.

### 5.4. AI and Machine Learning for Dental Adhesive Development

Machine learning (ML) and artificial intelligence (AI) will significantly advance the development of dental adhesives by optimizing formulations, accelerating testing, enhancing performance, and personalizing treatments. By leveraging existing data on methacrylate-based dental adhesives, materials informatics (MI) in dental materials research applies computational techniques, particularly ML, to analyze and predict material properties, streamlining the discovery and development of dental restorative materials like resin composites, glass ceramics, and luting cement. Utilizing supervised learning models, MI algorithms identify optimal chemical compositions and enhance bonding strength, durability, and biocompatibility properties. Despite its potential, MI faces challenges, including the complexity of synthesizing new materials and limited integration with manufacturing processes. Future efforts aim to combine MI with process informatics (PI) and autonomous systems to optimize material design and production, supported by open-access databases and advanced robotics [367,368]. AI- and ML-powered virtual testing methods can simulate and forecast adhesive behavior under various clinical conditions, minimizing the need for time-consuming trial-and-error experiments. AI-driven research can also uncover new bioactive or antibacterial agents that enhance tooth health and help prevent secondary caries [367]. Additionally, AI can enable the customization of adhesives to individual patient needs by considering factors such as tooth structure, material properties, and clinical performance [369]. This data-driven approach accelerates innovation, shortens development timelines, and ensures the production of more reliable, efficient adhesives tailored to the demands of modern digital dentistry workflows [369,370]. Future directions emphasize the creation of sustainable, open-access databases and leveraging autonomous systems to advance both material discovery and production [368].

## 6. Limitations and Conclusions

This review has several limitations. First, a significant portion of the evidence presented is derived from in vitro studies, which may not fully replicate the complex oral environment. For instance, laboratory studies have demonstrated the hydrolytic stability of TEG-DVBE-based resins and the antibacterial efficacy of silver nanoparticle-doped adhesives. However, long-term clinical trials need to validate these findings to assess their performance under real-world conditions. Second, the rapid pace of innovation in dental adhesives means that some emerging technologies, such as AI-driven adhesive development and bioinspired adhesives, are still in their early stages. While AI has shown promise in optimizing adhesive formulations and predicting clinical outcomes, its application in dentistry is limited by the lack of large, high-quality datasets and the potential for algorithmic bias [368]. Similarly, bioinspired adhesives, such as those mimicking mussel adhesion proteins, offer exciting possibilities but require further research to address challenges related to scalability and biocompatibility [359]. Third, the review primarily focuses on methacrylate-based adhesives dominating the current market. However, alternative materials, such as siloranes and thiourethanes, have shown potential for improving bond durability and reducing polymerization shrinkage. Future studies should explore these materials in greater depth, particularly in the context of digital dentistry and 3D-printed restorations. Finally, the review highlights the importance of functional monomers, such as 10-MDP, in enhancing bond strength and durability. However, the long-term stability of these monomers in the oral environment remains a concern, as hydrolytic degradation and enzymatic activity can compromise the adhesive interface over time [36]. Addressing these challenges will require a multidisciplinary approach, combining advances in materials science, digital technologies, and clinical research.

In conclusion, the development of dental adhesives reflects a dedication to continuous innovation and improved patient care, paving the way for more effective, durable, and predictable dental restorations in the future. By addressing the limitations outlined above and embracing emerging technologies, researchers and clinicians can further advance the field of adhesive dentistry, ultimately benefiting patients through enhanced oral health outcomes.

## Figures and Tables

**Table 1:** Generations of dental resin adhesives.

Generation	Chemical Composition and Technique Used	Bonding Mechanism and Bond Strength (MPa)	Challenges	Example Brands
First; (1950s) [40,41,65]	Glycerophosphoric acid dimethacrylate (NPG-GMA) containing resin; Etch and rinse.	Chemical bonding; 1–3	Poor clinical performance due to the high interfacial stress and thermal expansion	Sevriton Cavity Seal.
Second; (1970s) [41,44]	Polymerizable phosphonate in bis-GMA; Etch and rinse	Chemical bonding; 4–6	Prone to degradation and microleakage.	Scotchbond 1 (3M)
Third; (Late 1970s) [10,47]	Urethane dimethacrylate (UDMA), Bis-GMA, Primers (PENTA mix, HEMA, and ethanol), Mild acids; Etch and rinse, multi-step	Micro-mechanical interlocking and chemical bonding; 15–20	Unable to infiltrate the smear layer.	OptiBond FL (Kerr), Prime a Bond (Dentsply), Scotchbond Multi-Purpose (3M)
Forth; (late 1980s) [10,41]	Bis-GMA, Hydrophilic primer HEMA, Glass filler, Phosphoric acid etchant; Total-etch.	Micro-mechanical interlocking and chemical bonding; 20–25	Time-consuming, sensitive to technique and moisture control.	OptiBond FL (Kerr), Prime & Bond (Dentsply)
Fifth; (1990s) [10,51]	Bis-GMA, HEMA (merging primer and adhesive resin); One-step, total-etch.	Micro-mechanical interlocking; 20–30	Prone to water sorption and degradation overtime.	Single Bond (3M), Excite (Ivoclar Vivadent)
Sixth; (2000s) [10,51,53,66]	Addition of Silanes, adhesion promoters, mild acids; Self-etch, no rinse.	Micro-mechanical interlocking and chemical bonding; 25–30	Less durable bond than 4th/5th generations.	Adper Prompt (3M), Clearfil SE Bond (Kuraray)
Seventh; (2010s) [10,56,57]	All-in-one adhesives, various methacrylate, and HEMA; One-step, self-etch	Micro-mechanical interlocking and chemical bonding; 25–30	Lower cross-linking, higher hydrophilicity, decreased polymerization, polymer plasticization, potential allergic reactions, oxidative stress, and cytotoxicity.	G-Bond (GC), iBond (Heraeus Kulzer)
Eighth; (Recent) [10,67]	Functional monomers (e.g., 10-MDP, PENTA and GPMD); both etch-and-rise and self-etch strategies	Micro-mechanical interlocking and chemical bonding; 35–40+	Complexity in choice and application methods.	Universal Bond (3M), Tetric EvoFlow (Ivoclar Vivadent)

**Table 2:** Key differences between the 7th and the 8th generations of resin adhesives: [2,25,56,68,69,70,71].

Aspect	7th-Generation Adhesives	8th-Generation Adhesives
Application mode	Self-etch only	Multi-mode (se, er, selective etch)
Bond strength	Moderate, weaker on enamel	Superior, especially in er mode
Technique sensitivity	High (moisture-sensitive)	Low (more forgiving)
Durability	Prone to hydrolytic degradation	Improved resistance to degradation
Versatility	Limited to self-etch approach	Compatible with multiple substrates and modes
Monomer technology	Basic acidic monomers (e.g., MDP, 4-META)	Advanced monomers (e.g., 10-MDP)

**Table 3:** Most common existing commercially available resin formulations [2,25,56,68,69,70,71].

Resin Network	Properties Improved	Drawbacks	Reference
Bis-GMA/HEMA	Aesthetics, handling, high bond strength, flexibility, and stress distribution.	Biologic safety of bisphenol A. HEMA leaching and water degradation.	[42,77,78,79,80,81,82,83,84,85,86,87]
Bis-GMA/TEGDMA	Good handling, chemical stability, and strong acrylic bonds with inorganic fillers.	High water sorption, reduced mechanical properties, and low color stability.	[86,87]
UDMA/HEMA	Higher FS, EM and hardness as well as improved monomer conversion compared to Bis-GMA.	HEMA leaching and water degradation.	[85,86,87,88]
UDMA/HEMA/4-MET	Addition of 4-MET significantly increased mean SBS to dentin.	HEMA leaching and water degradation.	[89]
UDMA/TEGDMA	Higher FS, EM and hardness as well as improved monomer conversion compared to Bis-GMA.	High water sorption, reduced mechanical properties, and low color stability.	[86,87,88]
Bis-GMA/TEGDMA/UDMA	Improved the overall degree of conversion and mechanical properties.	Biologic safety of bisphenol A. High water sorption.	[86,87,88,90]
UDMA/Bis-GMA/HDDMA	Lowers the overall viscosity of the composite, allowing more filler or additional components.	Low FS and FM	[91]
UDMA/GDMA	Improve mechanical properties, hydrolytic resistance and reduce cytotoxicity.	GPDM-Ca salts are more prone to hydrolytic degradation than other functional monomers.	[9,92,93]
UDMA/Bis-GMA/TEGDMA	Acceptable mechanical properties.	Biologic safety of bisphenol A.	[91]
UDMA/Bis-GMA/TEGDMA/HEMA	Improved mechanical properties, such as hardness, TBS, FS and FM, and lower shrinkage stress compared to Bis-GMA.	Biologic safety of bisphenol A. HEMA leaching and water degradation.	[91]
UDMA/Bis-GMA/HEMA	Acceptable mechanical properties.	Biologic safety of bisphenol A. HEMA leaching and water degradation.	[91]
Bis-GMA/HEMA/GPDM	Promotes adhesive diffusion into the demineralized dentin and forms an instable GPDM-Ca salt with hydroxyapatite.	GPDM-Ca salts are more prone to hydrolytic degradation compared to other functional monomers, e.g., 10-MDP.	[9,94]
Bis-GMA/HEMA/4-META	4-Meta is a functional monomer that forms 4-META-Ca salt with the hydroxyapatite.	Faster solubilization of 4-META-Ca compared to 10-MDP-Ca, resulting in lower molecule stability.	[4,94,95,96]
Bis-GMA/HEMA/10-MDP	Remarkable bond strength and longevity with 10-MDP-based adhesives.	Chemical interaction with HAp crystals in unetched enamel is less effective than in dentin.	[8,94,96,97,98,99,100,101,102,103]

**Table 4:** Experimental resins to advance dental adhesives.

Resin Network	Properties Improved	Drawbacks	Reference
Bis-GMA /HEMA/Riboflavin and D-Alpha 1000 Succinate polyethylene (VE-TPGS)	Facilitate resin penetration in dentine and the distribution and uptake of riboflavin through extracellular and collagen matrices. VE-TPGS effectively quenches harmful reactive-oxygen species.	Long-term clinical studies are required to validate these findings.	[180,181]
HEMA/Bis-GMA/TMPEDMA	TMPEDMA improved the esterase resistance.	Long-term clinical studies are required to validate these findings.	[182]
BCF-EA/TEGDMA (5E5T) BCF-EA/TEGDMA (5G5T)/1 wt% DMAEMA	Derived from renewable bio-based raw materials Lower cytotoxicity.	Long-term clinical studies are required to validate these findings.	[183]
4-TF-PQEA/TEGDMA	Lower polymerization shrinkage, water sorption, and higher DC values compared to Bis-GMA/ TEGDMA resin system.	Lower mechanical properties.	[184]
SiMA/TEGDMA/Silanization of BaAlSiO2 microfillers. /0.7 wt% DMAEMA	Reduced human exposure to Bisphenol A derivatives.	Mechanical properties of SiMA based resins need improvement, and further research on their biocompatibility is required.	[185]
UDMA/SiMA/TEGDMA	Eliminates bisphenol A derivatives in the oral environment. Higher DC, less shrinkage, comparable FM, lower WS, and water solubility compared to Bis-GMA/TEGDMA	Further research is needed to optimize resin formulations and assess biocompatibility.	[185]
UXY modified urethane resin/HDDMA	New aliphatic and aromatic urethane dimethacrylate monomers containing pendant phenyl methoxy significantly reduced water sorption and water solubility of urethane based dimethacrylate systems.	Further studies are needed to evaluate the bonding values and other mechanical properties.	[186]
TMBPF-Ac or TMBPF-Ac/TEGDMA	Eliminates bisphenol A derivatives in the oral environment and exhibits superior mechanical properties and lower cytotoxicity compared to Bis-GMA/TEGDMA formulations.	The in vitro results are promising, but extensive long-term clinical studies are required to validate these findings.	[187]
Polymerizable collagen cross-linker methacrylate-functionalized proanthocyanidins (MAPA): MAPA-1, MAPA-2, and MAPA-3/0.5 wt% CQ/EDMAB/DPIHP	MAPA is a novel collagen cross-linker that stabilizes dentin collagen and improves polymerization, mechanical properties, and stability of HEMA-based adhesives.	Further research is needed to evaluate the effects of MAPA in commercial adhesives and as a primer in clinical settings.	[188,189]
Bis-GMA/QAUDMA-m/TEGDMA	QAUDMA-m demonstrates good mechanical performance and high antibacterial activity against *S. aureus* and *E. coli*.	The in vitro results are promising, but long-term clinical studies must validate these findings.	[190]
2EMATE-BDI/UDMA	Bis-GMA-free dental resin composites reduce polymerization stress without compromising mechanical properties while maintaining hydrophobicity and minimizing biofilm formation and stress.	Further studies are needed to balance this new monomer with other antibacterial monomers to reduce biofilm formation and improve the longevity of dental composite restorations.	[191]
A series of three nanogels: NG1—IBMA/UDMA; NG2—HEMA/Bis-GMA; NG3—HEMA/TE-EGDMA. That are dispersed in solvent, HEMA or Bis-GMA/HEMA.	Nanogels with varying hydrophilicity influenced mechanical performance and dentin bond strength. Generally, the more hydrophobic IBMA/UDMA nanogel exhibited better bulk material mechanical properties.	The in vitro results are promising, but extensive long-term clinical studies are required to validate these findings.	[192]
Dual Peptide Tethered Polymer: K-GSGGG-HABP: AMPM7 Polymer.	AMPM7 exhibited antimicrobial activity, and HABP provided peptide-mediated remineralization and high mineral binding properties.	Lack of mechanical and physical properties testing. Future studies must address the long-term retention of the antimicrobial activity under relevant in vivo conditions.	[193]
Bis-GMA/HMFBM	Comparable flexural strength and degree of conversion, low volumetric contraction excellent and cellular viability of fibroblasts.	The in vitro results are promising, but extensive long-term clinical studies are required to validate these findings.	[194]
UDMA/TEG-DVBE (U/V) PMGDM/TEG-DVBE (P/V)	However, TEG-DVBE-containing adhesives showed comparable shear and tensile bonding strengths to the dentin and resin composites, with superior stability after thermocycling. This performance was linked to improved mechanical properties, better dentin infiltration, and reduced water sorption/solubility.	Further studies are needed to evaluate the curing characteristics, polymerization shrinkage, and in vitro release of unreacted substances from the selected urethane monomers.	[195]
Multi-functional acrylamides: DEBAAP/UDMA/BMAAPMA TMAAEA/UDMA/BMAAPMA BAADA/UDMA/BMAAPMA UDMA/BMAAPMA	Interfacial bond strength was more significant and stable in the long term than methacrylate. (less than 4% reduction vs. 42% reduction in 6 months). HEMA degraded by almost 90%, while the acrylamides showed no degradation in acidic conditions.	Compared to methacrylate, these acrylamides had a lower overall degree of polymerization conversion. Long-term clinical studies are needed to validate these findings.	[196,197]
TDDMMA/TEGDMA	Bisphenol-A is free, with higher double bond conversion, lower solubility, and better mechanical properties after water immersion compared to Bis-GMA.	Higher water sorption. Further research is needed to investigate biocompatibility and resistance to oral microbial attachment.	[198]
FDMA/TEGDMA	FDMA-based resin had several advantages over Bis-GMA-based resin, such as higher double bond conversion, lower volumetric shrinkage, and better water resistance.	FDMA-based resin has higher viscosity than Bis-GMA-based. Further research is needed on biocompatibility and resistance to oral microbial attachment.	[199,200,201]
FDMA/FBMA FDMA/TEGDMA	Fluorinated methacrylate-based resin reduced *S. mutans* adhesion, with higher double bond conversion and lower water sorption and solubility than Bis-GMA/TEGDMA.	The in vitro results are promising, but long-term clinical studies must validate these findings.	[200]
FUDMA/TEGDMA/5 wt% of bioactive glass fillers	Bis-GMA free dental resin, with improved physicochemical properties	The in vitro results are promising, but extensive long-term clinical studies are required to validate these findings.	[202,203]
Urushiol derivative/HEMA	Urushiol is a natural renewable monomer and is Bis-GMA free.	Mechanical and adhesive properties need to be improved.	[204]
Bis-GMA/TEGDMA/SiO_2_ nanofiber fillers	Improved mechanical properties, especially for the composite resin fillings by increasing the wear resistance and lowering polymerization shrinkage.	The in vitro results are promising, but extensive long-term clinical studies are required to validate these findings.	[205]

**Table 5:** Properties of experimental functional fillers and additives in dental adhesives.

Functional Filler/Additive	Added Benefit
Carbon nanoparticles	Increase bond strength and improve mechanical properties [278,279].
Calcium phosphate nanoparticles (cap)	Helps regenerate hydroxyapatite at the adhesive interface, improving dentin remineralization and reducing secondary caries risk [281,295].
Chitosan	Increase bond strength [296], reduce dentin permeability [297], and enhance adhesive antibacterial properties.
Chlorhexidine	Helps maintain bond strength by inhibiting enzymatic degradation [298,299].
Copper nanoparticles	Reduce bacteria and biofilm [222,282,283,284,285], inhibit MMP activity [282], reduce degradation of adhesive [284], and increase bond strength [222,285]
Hydroxyapatite	Reduces post-operative sensitivity [300,301], stabilizes the adhesive-dentin interface, and improves the bonding durability [286,287].
Doxycycline	Inhibit MMP activity [288] and bacterial growth, improving the adhesives longevity [288].
Iron oxide nanoparticles	Improve bond strength and mechanical performance [289,290].
Silver nanoparticles	Increase antibacterial activity by preventing bacterial adhesion and biofilm formation [293,302,303,304,305,306,307,308].
Zinc oxide nanoparticles	Inhibit bacteria and biofilm formation, strengthen the hybrid layer, and reduce enzymatic degradation [282,291,292].
Titania nanoparticles	Improves interfacial bond strength [309], inhibits bacterial growth [309,310], and improves physicochemical properties [310].
Boron nitride nanotubes (bnnts)	Enhance mineral deposition and bonding durability without compromising biocompatibility [311].
Graphene nanoplatelets	Prevent secondary caries while maintaining bond strength; currently undergoing long-term effectiveness testing [312].
Bis(methacryloyl)imidazolium ntf2 (bmi.ntf2)	Acts as an ionic liquid additive, reducing polymerization stress and enhancing the mechanical properties of adhesives [313]. It also exhibits antibacterial properties [314].
Thio-urethane monomer	Reduces polymerization shrinkage and enhances fatigue resistance.
Quaternary ammonium compounds (qacs), e.g., mdpb	Provides antibacterial properties to inhibit bacterial growth at the adhesive interface [315,316] without relevant changes in physicochemical and mechanical properties [307].
Pre-reacted glass ionomer (prg) fillers	Enhance bonding durability and prevent secondary caries by strengthening dentin through ion uptake in fluoride-releasing adhesives [312].
Wollastonite	Calcium silicate (CaSiO_3_) enhances dental adhesives by improving mechanical properties, promoting mineral deposition, and maintaining bonding stability over time [317].
Other materials	Increase antibacterial activities—benzyldimethyldodecyl ammonium chloride [318], eugenyl methacrylate [319], nisin [269], tt-farnesol [320], pyrogallol (py), polyhexamethylene guanidine hydrochloride [321], and triclosan [322]. Improve bond strength—Resveratrol [323], and 4-formylphenyl acrylate [324].

**Table 6:** Market-available dental adhesives that incorporate remineralizing agents, antibacterial properties, or functional innovations.

Function	Adhesive	Added Benefit
Reminealizing Dental Adhesives.	Clearfil SE Protect (Kuraray Noritake)	Contains MDPB (12-methacryloyloxy-dodecyl pyridinium bromide), which has antibacterial and remineralizing properties. Releases fluoride to aid in remineralization of demineralized dentin Demonstrates effectiveness in reducing secondary caries risk [327,328,329,330,331].
	OptiBond™ FL (Kerr)	Three-step etch-and-rinse adhesive with proven long-term clinical performance. Contains fluoride-releasing fillers to promote remineralization and reduce secondary caries risk. Provides excellent bond strength to enamel and dentin, even in challenging conditions. Features a hydrophobic resin layer that enhances sealing and reduces microleakage. Demonstrates high resistance to degradation in the oral environment [328,329].
	One-up Bond F (Tokuyama).	A Self-etching, fluoride-releasing adhesive improving bond durability and demineralization resistance [330,332].
Antibacterial Dental Adhesive	GLUMA Bond Universal (Heraeus Kulzer)	Contains 5% glutaraldehyde, which has antibacterial properties. Helps to reduce post-operative sensitivity while preventing bacterial growth at the adhesive interface. Designed for universal use in direct and indirect restorations [331].
	Prime & Bond Active (Dentsply Sirona)	Incorporates antibacterial monomers and moisture-tolerant chemistry. Demonstrates strong adhesion even in moist environments. Reduces the risk of microleakage and bacterial infiltration [333,334].
	Peak Universal Bond (Ultradent)	Contains chlorhexidine, which has antimicrobial effects to inhibit bacterial growth. Provides high bond strength to enamel and dentin. Compatible with both direct and indirect restorations [331].
	G2-BOND Universal (GC)	Contains quaternary ammonium compounds that disrupt bacterial cell membranes. Provides long-lasting antibacterial effects. Suitable for a wide range of restorative procedures [335,336].
Functionalized And Innovative Dental Adhesives	Bioactive iBOND Universal (Kulzer)	Features bioactive fillers that release calcium and phosphate ions. Promotes natural remineralization and enhances the durability of adhesive bonds. Used in both restorative and luting procedures [337,338].
	ACTIVA BioACTIVE Cement (Pulpdent)	Biomimetic properties—actively interact with the tooth structure. Releases calcium, phosphate, and fluoride to support natural remineralization. High fracture toughness and elasticity, mimicking natural dentin [294,339].
	G-Premio BOND (GC)	Nanotechnology-based adhesive with improved cross-linking density. Hydrophobic properties help improve longevity and bond strength. Improved polymerization stability, reducing long-term degradation [340,341,342].

## Data Availability

No new data were created or analyzed in this study. Data sharing is not applicable to this article.

## Appendix A. Acronym and Abbreviation List

10-MDP: 10-methacryloyloxydecyl dihydrogenphosphate A174: γ-methacryloxypropyltrimethoxysilane Coupling factor A174: g-methacryloxypropyltrimethoxysilane 2EMATE-BDI: 2-hydroxy-1-ethyl methacrylate 4-AETA: 4-acryloyloxyethyl trimellitate anhydride 4-AET: 4-acryloylethyl trimellitic acid 4-META: 4-methacryloyloxyethyl trimellitate anhydride 4-MET: 4-methacryloyloxyethyl trimellitic acid AMPS: 2-acrylamido-2-methyl-1-propanesulfonic acid BAADA: N,N′-bis(acrylamido) 1,4-diazepane Bis-EMA: ethoxylated bisphenol A glycol dimethacrylate Bis-GMA: bisphenol A diglycidyl methacrylate Bis-MEP: bis[2-(methacryloyloxy)ethyl] phosphate BMAAPMA: N,N-Bis[(3-methylaminoacryl)propyl]methylamine BPDM: biphenyl dimethacrylate or 4,40-dimethacryloyloxyethyloxycarbonylbiphenyl-3,30-dicarboxylic acid BPO: benzoyl peroxide (redox initiator) BS acid: benzenesulfinic acid sodium salt (redox initiator) CQ: camphorquinone or camphoroquinone or 1.7.7-trimethylbicyclo-[2,2,1]-hepta-2,3-dione (photo-initiator) DC: Double bond conversion DEBAAP: N,N-Diethyl-1,3-bis(acrylamido)propane Di-HEMA phosphate: di-2-hydroxyethyl methacryl hydrogenphosphate DMAEMA: dimethylaminoethyl methacrylate EAEPA: ethyl 2-[4-(dihydroxyphosphoryl)-2-oxabutyl]acrylate EGDMA: ethyleneglycol dimethacrylate EM: Elastic modulus F-PRG: full reaction type pre-reacted glass-ionomer fillers FBMA: fluorinated diluent 1 H,1 H-heptafluorobutyl methacrylate FDMA: fluorinated dimethacrylate FM: Flexural modulus FS: Flexural strength GDMA: glycerol dimethacrylate GPDM: glycerol phosphate dimethacrylate HDDMA: 1,6-hexanediol dimethacrylate HEMA: 2-hydroxyethyl methacrylate IBMA: isobornyl methacrylate MA: methacrylic acid MAC-10: 11-methacryloyloxy-1,10-undecanedicarboxylic acid MAEPA: 2,4,6 trimethylphenyl 2-[4-(dihydroxyphosphoryl)-2-oxabutyl]acrylate MDPB: methacryloyloxydodecylpyridinium bromide MF8P: 6-methacryloxy-2,2,3,3,4,4,5,5-octafluorohexyl dihydrogen phosphate NaF: sodium fluoride Na_2_SiF_6_: disodium hexafluorosilicate NPG-GMA: N-phenylglycine glycidyl methacrylate NTG-GMA: N-tolylglycine glycidyl methacrylate or N-(2-hydroxy-3-((2-methyl-1-oxo-2-propenyl)oxy)propyl)-N-tolyl glycine PEGDMA: polyethylene glycol dimethacrylate PEM-F: pentamethacryloyloxyethylcyclohexaphosphazene monofluoride PENTA: dipentaerythritol pentaacrylate monophosphate Phenyl-P: 2-(methacryloyloxyethyl)phenyl hydrogenphosphate PMDM: pyromellitic diethylmethacrylate or 2,5-dimethacryloyloxyethyloxycarbonyl-1,4-benzenedicarboxylic acid PMGDM: pyromellitic glycerol dimethacrylate or 2,5-bis(1,3-dimethacryloyloxyprop-2-yloxycarbonyl)benzene-1,4-dicarboxylic acid POSS nano-particulates: polyhedral oligomer silsesquioxanes QAUDMA-m: quaternary ammonium urethane-dimethacrylate derivative (QAUDMA-m, where m was 8, 10, 12, 14, 16, 18, and corresponded to the number of carbon atoms in the N-alkyl substituent) SBS: Shear bond strength SiO_2_ nanofiber fillers: silicon dioxide nanofibers TBS: Tensile bond strength TE-EGDMA: tetraethylene glycol dimethacrylate TEG-DVBE: triethylene glycol divinylbenzyl ether TEGDMA: triethylene glycol dimethacrylate TMAAEA: Tris[(2-methylaminoacryl)ethyl]amine TMPTMA: trimethylolpropane trimethacrylate UDMA: urethane dimethacrylate or 1,6-di(methacryloyloxyethylcarbamoyl)-3,30,5-trimethylhexane
VS: Volumetric shrinkage WS: Water sorption WSL: Water solubility
